# The biological function of *Atractylodes lancea* and its application in animal husbandry: a review

**DOI:** 10.3389/fvets.2024.1518433

**Published:** 2025-01-10

**Authors:** Yang Gao, Dong Wang, Xue Ma, Jiahui Li, Difei Wang, Bo Chen, Xuexi Yang, Huan Leng

**Affiliations:** ^1^College of Life Science, Baicheng Normal University, Baicheng, China; ^2^College of Veterinary Medicine, Northwest A&F University, Xianyang, Shaanxi, China; ^3^Terra Research and Teaching Centre, Microbial Processes and Interactions (MiPI), Gembloux Agro-Bio Tech, University of Liège, Gembloux, Belgium; ^4^Key Laboratory of Development and Application of Rural Renewable Energy, Biogas Institute of Ministry of Agriculture and Rural Affairs, Chengdu, China

**Keywords:** *Atractylodes lancea*, biological functions, application, animal husbandry, green cultivation

## Abstract

*Atractylodes lancea*, is a herbaceous plant of the Asteraceae family which is a traditional Chinese herbal medicine. It is often used for dehumidification, antiemetics, spleen strengthening and antipyretic effects. *Atractylodes lancea* is rich in various bio-active substances and has many biological functions, for instance anti-inflammatory, antioxidant and antiviral effects. Therefore, it is widely used in animal production, such as relieving heat stress, protecting intestinal health and regulating immunity. In recent years, it has received widespread attention in green cultivation. This article reviews the biological functions of *Atractylodes lancea* and looks forward to its application prospects in animal husbandry, in order to provide a theoretical basis for *Atractylodes lancea* to become a new feed additive in animal production.

## 1 Introduction

*Atractylodes lancea* (*A. lancea*) is a perennial herb with a light aroma. It is often used for dehumidification, antiemetics, spleen strengthening and antipyretic effects (1–3), as well as relieve pain and diarrhea (4). In recent years, it is widely used to treat vomiting (5) and heatstroke (6), which is one of the traditional Chinese herbal medicine in China. The rhizome of *A. lancea* has been used widely in many countries for various indications. This compound is called “Cangzhu” in China, “Khod-Kha-Mao” in Thailand, and “So-jutsu” in Japan. There are many species of *A. lancea*, which are widely distributed around the world. Studies have found that *A. lancea* has many biological functions, including anti-inflammatory, antioxidant, antiviral, antibacterial, analgesic, so that it can be used for improving gut health, immunity and growth performance of animals (7, 8). Nowadays, with the continuous development and research of new alternative antibiotic products, *A. lancea* and its extracts have been widely used in animal husbandry due to its low cost, high efficiency and low toxicity. This article reviews the main active ingredients and biological functions of *A. lancea*, and looks forward to its application prospects in livestock and poultry production, in order to provide a reference for the efficient use of *A. lancea* in green and healthy cultivation.

## 2 The main biological compounds and their structures of *A. lancea*

**Figure 1:**
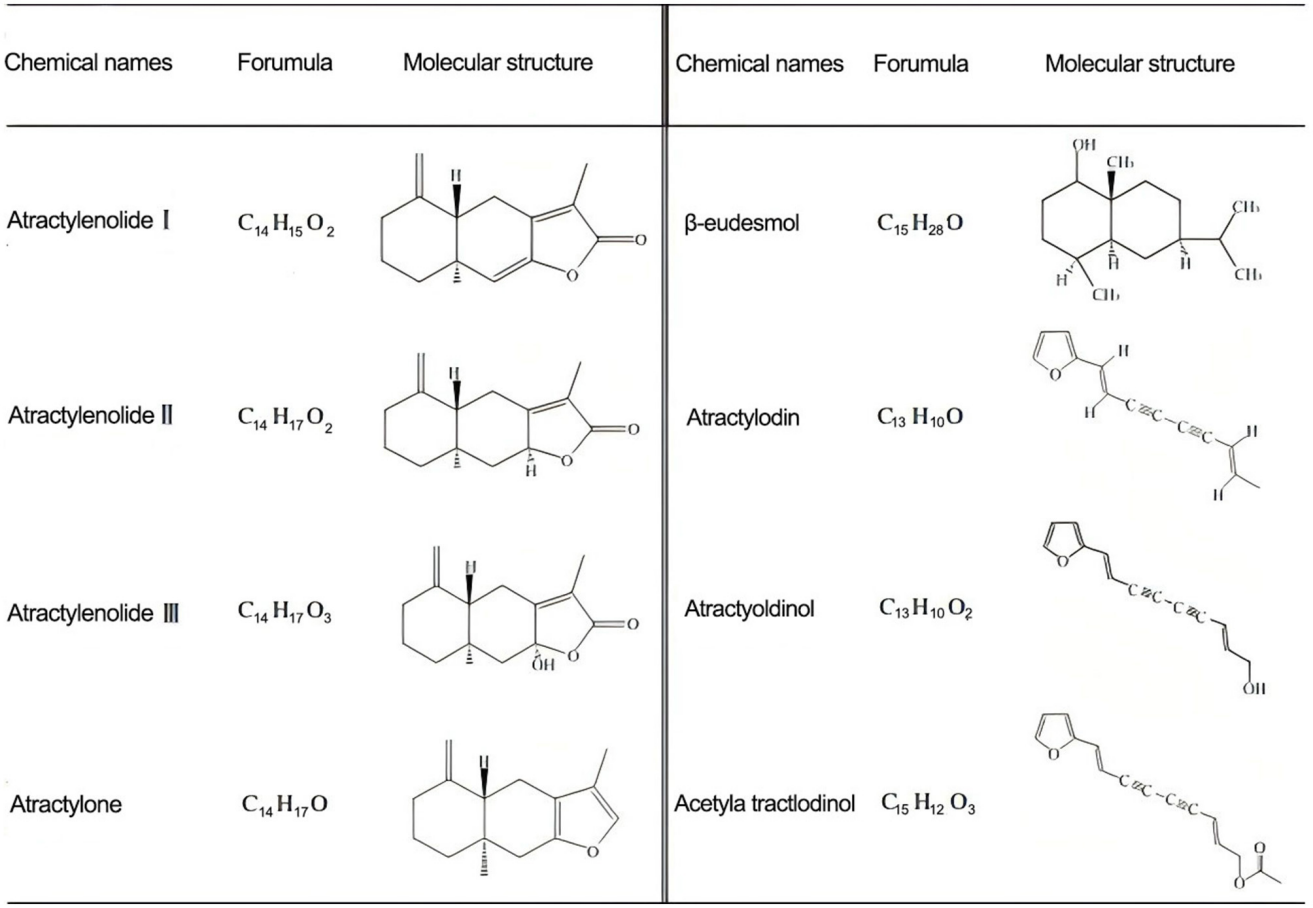
The main bio-active compounds of *A. lancea* and their structures.

*lancea* has a variety of natural bio-active compounds, including sesquiterpenes, enynes, aromatics, polysaccharides, flavonoids, phenols and organic acids (9). The main components of *A. lancea* are sesquiterpenes, including atractylodin, atractylone, β-eudesmol, atractylodes polysaccharides, and atractylenolide (10). The content of active ingredients in *A. lancea* was analyzed by gas chromatography-mass spectrometry (GC-MS). The results showed that the content was as follows: atractylodin (6.22%), hinesol (3.52%), atractylone (1.32%), β-eudesmol (0.81%), and atractylol (0.15%) (11). Wang et al. used a variety of methods to separate the volatile oil of *A. lancea* and found that the contents of atractylodin, atractylol, and atractylone were high, while the contents of atractylenolide and atractylodes polysaccharides were low. At the same time, it was confirmed that *A. lancea* has good pharmacological activities (12). The main bio-active compounds of *A. lancea* and their structures are shown in Figure 1.

## 3 The biological functions of *A. lancea*

### 3.1 Anti-inflammatory

Inflammatory response is a complex physiological response of the immune system to external stimuli, which is regulated by a variety of inflammatory mediators. Appropriate amount of inflammatory factors plays an important role in maintaining the normal physiological functions of animals, but when animals are infected by viruses or bacteria, a large amount of inflammatory factors will be deposited in the body, leading to serious inflammatory damage (13–15). Atractylenolide I and atractylenolide III can reduce the expression level of tumor necrosis factor-α (TNF-α) and the production of nitric oxide (NO) in animal serum. Atractylenolide I showed a more effective inhibitory effect on the production of TNF-α and NO in peritoneal macrophages activated by lipopolysaccharide (LPS) than atractylenolide III (16). Both nuclear factor-κB (NF-κB) and mitogen-activated protein kinase (MAPK) signaling pathways can regulate the release of inflammatory factors in animals. The study by Jeong et al. (17) showed that atractylenolide I and Atractylenolide III could inhibit the phosphorylation of p38 MAPK, c-Jun N-terminal kinase (JNK), and inhibitor of nuclear factor-κB (IκB) in LPS induced mouse inflammatory macrophages, promote the phosphorylation of extracellular signal-regulated kinase (ERK), block the translocation of NF-κB to the nucleus, and significantly reduce the expression of pro-inflammatory factors such as TNF-α, interleukin-6 (IL-6), and interleukin-1β (IL-1β). In addition, atractylenolide II can reduce the release of inflammatory factors such as NO, TNF-α, and IL-6 in mouse macrophages induced by LPS, but the effect is not significant (18). In summary, atractylenolide, as the most effective anti-inflammatory component in *Atractylodes lancea*, can reduce the expression level of inflammatory factors and alleviate inflammatory damage by regulating the MAPK and NF-κB signaling pathways, but the inflammatory regulation mechanism of other compounds still needs further study.

### 3.2 Antioxidant

Reactive oxygen species (ROS) are extremely bio-active materials. Excessive levels of ROS in livestock and poultry can cause oxidative stress (19). The phenolic acids and flavonoids contained in *Atractylodes lancea* have metal chelating and free radical scavenging functions, inhibiting the production of ROS (20). The Nrf2-Keap1 signaling pathway plays a pivotal role in the antioxidant response of animals and increase the activity of antioxidant enzymes, among which Nrf2 is the main effector (21). Study had shown that Atractylodes polysaccharide II can increase the activity of glutathione peroxidase (GSH-Px) and superoxide dismutase (SOD) in the tissue of mouse liver, significantly reduce the activity of nitric oxide synthase (NOS) and the content of NO and malondialdehyde (MDA) (22). It can be seen that *A. lancea* polysaccharide has good antioxidant activity. On the one hand, *A. lancea* polysaccharide balances the antioxidant system in animals by inhibiting the production of ROS; On the other hand, *A. lancea* polysaccharide can regulate the Nrf2-Keap1 signaling pathway, enhance the activity of antioxidant enzymes SOD and GSH-Px, and improve the ability of animals to clear ROS.

### 3.3 Anti-viral

Viral infections such as swine flu and avian flu are extremely harmful to livestock and poultry production. The clinical manifestations include diarrhea, cough, fever, and lameness. Mild cases can cause breathing and movement difficulties, while severe cases can lead to large-scale deaths, seriously affecting the economic benefits of the breeding industry. *A. lancea* can act on the cell surface to change the protein receptor structure, significantly block the adsorption and penetration of swine influenza virus into cells. Study has shown that *A. lancea* can significantly inhibit the proliferation of swine influenza virus in cells and has a direct effect of inactivating virus (23, 24). In addition, atractylone, as the main antiviral ingredient in *A. lancea*, can block the adsorption and replication of avian influenza virus (25). Toll-like receptor 7 (TLR7) is widely present in immune cells, epithelial cells and nerve cells. When livestock and poultry are invaded by pathogens, TLR7 will immediately transfer to the cell membrane, identify the pathogens, aggregate specific proteins to activate regulatory factors such as IκB, MAPK and interferon (IFN), initiate specific immune responses, and participate in the antiviral process. Atractylone can regulate TLR7 receptors, inhibit the activation of MAPK and NF-κB signaling pathways, relieve epidemic diarrhea and respiratory diseases caused by influenza A virus infection. Chen et al. (26) found that continuous treatment with 10, 20, and 40 mg/kg atractylone for 5 days could alleviate influenza A virus-induced lung injury in mice, significantly reduce serum TNF-α, IL-6, and IL-1β levels, meanwhile increase IFN-β levels, indicating that atractylone can promote IFN-β production by activating the TLR7 signaling pathway, interfere with viral replication, and recruit immune cells to activate specific immune responses and eliminate viruses. Therefore, *A. lancea* has important application potential in livestock and poultry production because of its antiviral effects.

### 3.4 Other functions

**Figure 2:**
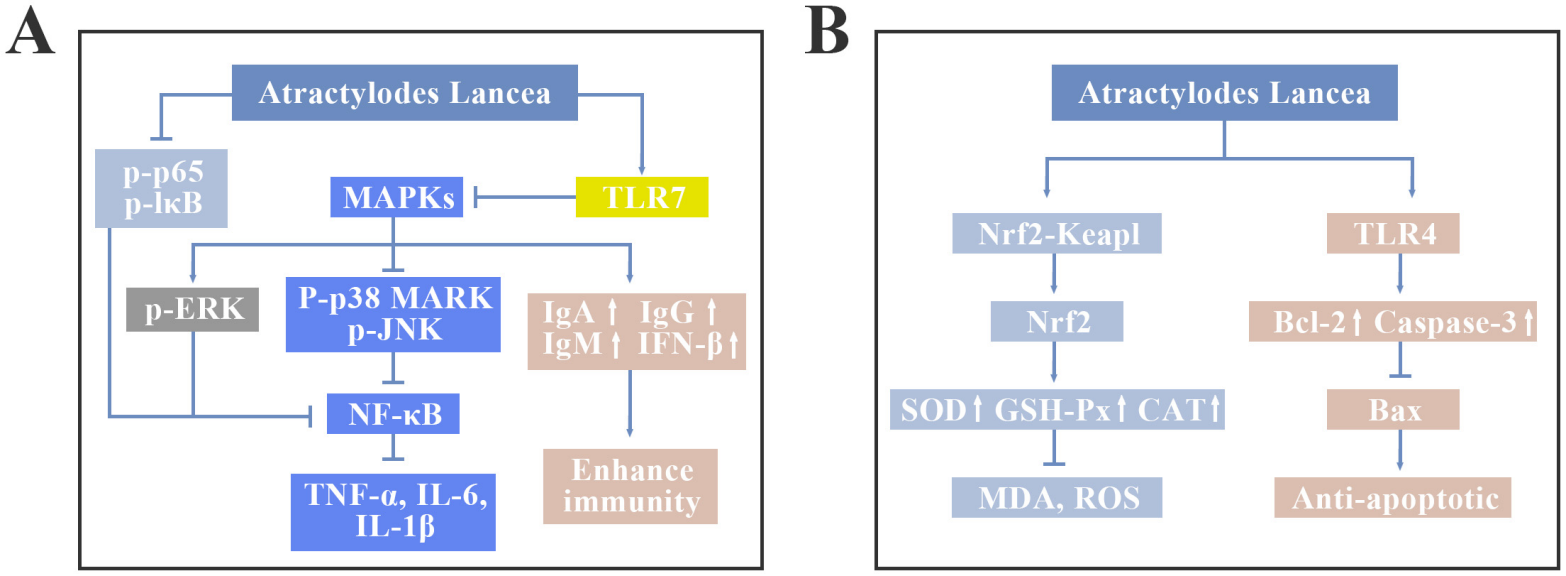
The regulatory mechanism of *A. lancea* on MAPK, NF-κB, Toll-like receptor (TLR) and Nrf2-Keap1 signaling pathways. **(A)** MAPK, NF-κB and TLR7 signaling pathways. **(B)** Nrf2-Keap and TLR4 signaling pathways.

*A. lancea* also has anti-tumor, liver protection, diuretic, glucose metabolism, and lipid metabolism regulating functions. Studies have shown that *A. lancea* polysaccharides have anti-tumor effects, which can activate macrophages through the Toll-like receptor 4 (TLR4) signaling pathway, reduce the expression of B cell lymphoma-2 (Bcl-2), increase the expression of pro-apoptotic factors such as Bcl-2-associated X protein (Bax) and cysteine aspartate proteinase-9 (Caspas-9), finally promote tumor cell apoptosis (27–29). Additionally, *A. lancea* polysaccharides can prevent liver damage caused by harmful chemicals and toxins. Han et al. (22) showed that *A. lancea* polysaccharide can reduce the expression levels of AST, ALT, and MDA in liver, increase the activity of SOD and GSH-Px, and alleviate LPS-induced liver inflammation in mice by inhibiting the NF-κB signaling pathway. It is reported that *A. lancea* has a diuretic effect. Study has found that intravenous injection or oral administration of 1.0 g/kg *A. lancea* solution can significantly increase the urine output of mice (27). In addition, the active ingredients in *A. lancea* can improve glucose uptake, inhibit fat production, and regulate lipid metabolism in animals (30). The regulatory mechanism of *A. lancea* on MAPK, NF-κB, Toll-like receptor (TLR) and Nrf2-Keap1 signaling pathways is shown in Figure 2. The key functions and associated mechanisms of *Atractylodes lancea* was showed in Table 1.

**Table 1:** The key functions and associated mechanisms of *Atractylodes lancea*.

**Biological functions**	**Active ingredients**	**Relative mechanisms**	**References**
Anti-inflammatory	Atractylenolide I	TNF-α, NO, IL-6, IL-1β↓	(16)
	Atractylenolide III	p38 MAPK, JNK, IκB↓	(17)
			(18)
Antioxidant	Atractylodes polysaccharide II	GSH-Px, SOD↑	(22)
		NOS, NO, MDA↓	(20)
Anti-viral	Atractylone	MAPK and NF-κB signaling pathways↓	(25)
		TNF-α, IL-6, IL-1β↓	(26)
Anti-tumor	Atractylodes polysaccharides	Bcl-2↓	(27–29)
		Bax, Caspas-9↑	
Liver protection	Atractylodes polysaccharides	AST, ALT, and MDA↓	(27)
		SOD, GSH-Px↑	

## 4 Application prospects of *A. lancea* in livestock and poultry production

### 4.1 Improving productive performance

*A. lancea* is rich in natural active substances, which have multiple functions such as improving immunity, resisting stress and promoting digestion. It has great potential in improving the production performance of livestock and poultry. Li et al. (31) found that adding 3, 6, and 9 g/kg of *A. lancea* polysaccharide to the diet could significantly improve the growth performance of early weaned piglets. On the one hand, *A. lancea* polysaccharides can play an antioxidant role and alleviate the adverse effects of stress on growth performance (32). On the other hand, *A. lancea* polysaccharide can stimulate lymphocyte proliferation and antibody production in early weaned piglets, improve disease resistance and reduce diarrhea rate (33). In addition, *A. lancea* also plays an important role in improving the productive performance of poultry and ruminants. Study has found that adding *A. lancea* polysaccharides to laying hen diets can significantly increase egg weight, egg production rate, and feed conversion rate, reduce mortality of laying hens (34). In the ruminants, studies have shown that adding 0.75% of *A. lancea* to the diet can improve the fermentation capacity, increase the efficiency of rumen microorganisms in degrading substances such as protein and cellulose, and promote the synthesis of bacterial protein in the rumen (35). It can be seen that *A. lancea* has good application value and research potential in improving the production performance of livestock and poultry, but its specific addition amount needs further exploration.

### 4.2 Immune regulation

After weaning, piglets lose the protection of maternal antibodies and their immune system is not fully developed, so they are easy to meet with problems such as poor disease resistance, growth retardation and diarrhea. Li et al. (36) added 0.3% purified *A.lancea* polysaccharide, 0.6% crude polysaccharide and 0.6% crude *A. lancea* polysaccharide to the diet of weaned piglets. The results showed that all three polysaccharides could increase the antibody content in serum, promote lymphocyte proliferation and improve immunity of weaned piglets. Wang et al. (37) found that adding 0.1, 0.2, and 0.3% fermented *A. lancea* to the diet of early weaned piglets could increase the levels of immunoglobulin A (IgA), immunoglobulin G (IgG) and immunoglobulin M (IgM) as well as TP and ALB in the serum, among which 0.2% fermented *A. lancea* had the most significant effect. Interleukin-1(IL-1) and interleukin-2 (IL-2) are cytokines produced by activated T cells, which can stimulate the proliferation and differentiation of immune cells and enhance animal immunity. The experimental results of Xu et al. (33) showed that adding an appropriate amount of *A. lancea* polysaccharide to the diet of weaned piglets can promote lymphocyte proliferation, increase the levels of antibodies such as IgA and IgG in serum and release cytokines such as IL-1 and IL-2, thereby improving the immunity of weaned piglets. *A. lancea* can significantly increase the spleen and thymus index of livestock and poultry, and it has a positive effect on improving immunity and intestinal health. Li et al. (38) found that *A. lancea* polysaccharide can alleviate cyclophosphamide-induced immune organ damage in geese. In ruminants, *A. lancea* polysaccharides can induce the proliferation of bovine mammary lymphocytes and activate immune cells (39). In summary, *A. lancea* can be used as an immunomodulator and has important application potential in livestock and poultry production, but its specific mechanism of regulating immunity needs further study.

### 4.3 Improve intestinal health

#### 4.3.1 Intestinal barrier

Intestinal health is an important factor affecting the production performance of livestock and poultry, it is also an important indicator for assessing animal welfare. Bose et al. (40) found that fermented *A. lancea* polysaccharide can alleviate LPS-induced intestinal epithelial cell damage and reduce intestinal mucosal permeability through *in vitro* experiments. Shi et al. (41) found that the *A. lancea* can promote the integrity of the intestinal mucosal barrier by inhibiting the phosphorylation of p38 and MAPK signaling pathways and increasing the mRNA expression of *ZO-1, Claudin-1*, and *Occludin*. This indicates that *A. lancea* protects the intestinal barrier of animals by reducing the level of inflammatory factors. In addition, adding an appropriate amount of *A. lancea* polysaccharide to the LPS-induced enteritis model can increase the protein level of ZO-1 and Occludin, and alleviate intestinal inflammation in goose (42). In terms of intestinal microbes, *A. lancea* polysaccharides can reduce the relative abundance of *Escherichia coli* in weaned piglets while increasing the level of *Lactobacillus* (36). In addition, Wang et al. (43) reported that *A. lancea* polysaccharides can regulate the structure of intestinal microbes, promote the colonization of probiotics in the intestine, and reduce the abundance of harmful bacteria in mice. Studies on ruminants have shown that adding *A. lancea* to the diet can increase the activity of rumen microbiota, changing the structure of rumen microbes, and promoting rumen fermentation (44, 45). It can be seen that *A. lancea* plays a regulatory role in the intestinal health of animals, but its role in affecting the composition and activity of intestinal microbes still needs further study.

#### 4.3.2 Colon health

Colitis often occurs in piglets from 4 to 16 weeks of age after weaning, leading to increasing intestinal mucosal permeability, diarrhea, and reduced the growth performance of piglets (46). Studies have found that *A. lancea* can increase the content of tight junction proteins, thereby reducing diarrhea caused by colitis in mice (3, 47, 48). The mechanism of *A. lancea* in relieving colitis is as follows: on the one hand, *A. lancea* can promote the secretion of mucin by goblet cells, increase the content of ZO-1 and Occludin, reduce intestinal mucosal permeability, and restore the normal physiological function of colon in piglets; On the other hand, *A. lancea* inhibits the phosphorylation of MAPK and NF-κB signaling pathways in the intestine, reduces the expression of inflammatory factors such as TNF-α, IL-6, and IL-1β, alleviates the absorption disorder of sodium ions and chloride ions disturbed by inflammatory factors, improves the water re-absorption capacity in the colon, and reduces diarrhea in piglets. IL-6, which is a reference indicator of colitis, can increase the permeability of intestinal mucosa (49). Studies have shown that *A. lancea* can inhibit the release of TNF-α and IL-6, up-regulate the expression of autophagy genes, and significantly alleviate colon damage in mice (3, 50, 51). Since colitis is a disease caused by immune system disorders, it is speculated that *A. lancea* can maintain the homeostasis of the internal environment by activating the cell autophagy pathway and ultimately improve colitis.

### 4.4 Relief of heat stress

Heat stress refers to the sum of non-specific physiological responses made by the body to any requirements imposed by the thermal environment at high ambient temperatures (52). In general, there is an isothermal zone in homeothermic animals, and when the ambient temperature is in the range of the isothermal zone, the animal can maintain normal temperature through body temperature regulation; When the ambient temperature is higher than the upper limit of the isothermal zone, the animal will be subjected to heat stress (64). When heat stress occurs, animals typically show increased breathing, increased heart rate, and impaired electrolyte balance (53). Therefore, heat stress can effect the growth performance and reproductive performance. Under the influence of heat stress, animals' appetite decreases, resulting in a decrease in feed intake, which seriously affects production performance (54). Studies have shown that heat stress can lead to oxidative stress, adding *A. lancea* to pig diets can increase the activity of antioxidant enzymes in serum, reduce MDA and ROS levels, and thus alleviate heat stress (55, 56). *A. lancea* can terminate lipid peroxidation by removing ROS and hydrogen peroxide, improve ROS-induced intestinal epithelial cell shedding, and restore the digestive and absorptive functions of the intestine. Xu et al. found that adding *A. lancea* polysaccharides to broiler diets can significantly improve the antioxidant capacity, enhance immunity, and alleviate the damage caused by heat stress (57). In addition, *A. lancea* also has a good effect in alleviating heat stress in ruminants. It has been reported that under heat stress conditions, adding *A. lancea* to cattle diets can significantly improve immunity and antioxidant capacity, promote rumen digestion and absorption of nutrients in the feed, and improve production performance (57). It can be seen that *A. lancea* plays an important role in alleviating heat stress in animals, but its effective components and optimal additive dosage need further study.

### 4.5 Other application prospects

#### 4.5.1 Antimicrobial agents

Studies have shown that *A. lancea* has antibacterial effects on a variety of microbes, including *Escherichia coli, Candida albicans* and *Staphylococcus aureus* (2, 58). Peng et al. reported that 5–40 mg/mL Atractyoldinol had a significant inhibitory effect on *Staphylococcus aureus, Escherichia coli*, and *Bacillus subtilis* (59). The antibacterial mechanism of *A. lancea* is that on the one hand, *A. lancea* can destroy the protein structure in the bacterial cell *membrane*, causing the cell contents to flow out; On the other hand, adding *A. lancea* to animal feed as a feed additive can directly remove aflatoxin and improve the digestibility of nutrients in the feed. It can be seen that *A. lancea* has important application value in livestock and poultry production as an antibacterial agent.

#### 4.5.2 New type of analgesic agents

**Figure 3:**
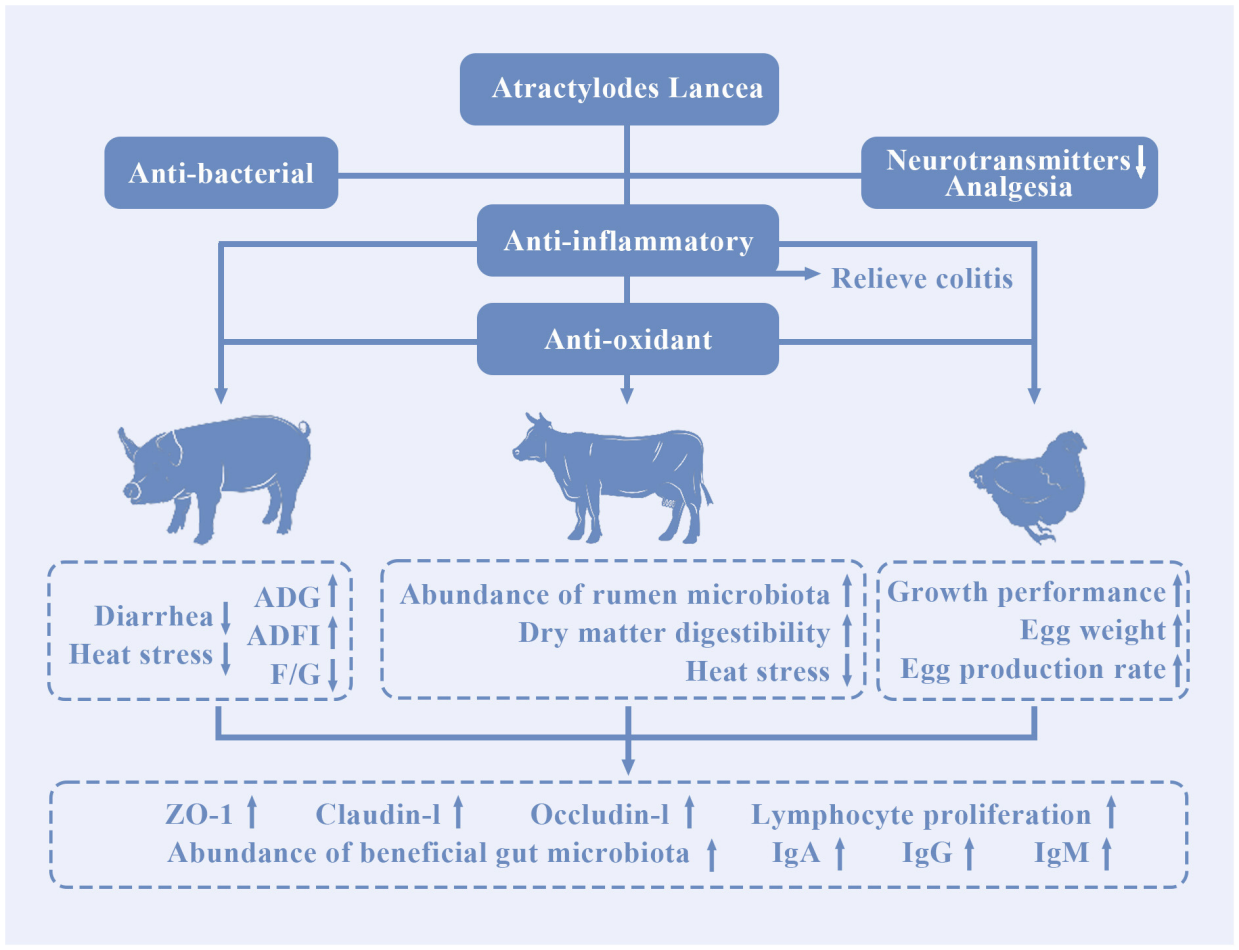
The application of *A. lancea* in livestock and poultry production.

Ohara et al. found that atractyoldinol and β-eudesmol have analgesic effects, which can inhibit the function of the nervous system, and reduce the sensitivity of animals to external stimuli (60). Atractylodesinol is used as an analgesic in livestock and poultry production. On the one hand, atractylodesinol can inhibit the release of neurotransmitters (61, 62); On the other hand, atractyoldinol can block acetylcholine receptor channels, weaken nerve signal transmission, and relieve pain caused by production processes such as sow farrowing, piglet castration and rectal prolapse suture (63). Therefore, atractyoldinol has a good analgesic effect which can be used as a new analgesic. However, it is currently rarely used in livestock and poultry, the specific dosage needs further experimental research to explore. The application of *A. lancea* in livestock and poultry production is shown in Figure 3.

## 5 Conclusion and perspectives

Under the background of “total ban on antibiotics,” optimizing livestock and poultry feed formula and finding new green feed additives have become research hotspots. *A. lancea* can inhibit the phosphorylation of MAPK and NF-κB signaling pathways, reduce the level of inflammatory factors, and alleviate the inflammatory response of livestock and poultry; At the same time, *A. lancea* can reduce the level of ROS and oxidative stress damage in animals by regulating the Nrf2-Keap1 signaling pathway; *A. lancea* can also inhibit the TLR signaling pathway to exert antiviral function. Adding *A. lancea* to animal diet can improve their growth performance and immunity, improve intestinal health and relieve heat stress. In addition, *A. lancea* can also be used as an antibacterial agent and analgesic, which has important application prospects in livestock and poultry production. However, the production process of *A. lancea* still needs to be optimized. Therefore, in the future, we should further develop *A. lancea* as a feed additive, promote its application in livestock and poultry production, explore the appropriate amount of addition at different growth stages, so as to promote the development of green ecological farming.

## References

[1] Yamahara J Matsuda H Huang Q Li Y Fujimura H. Intestinal motility enhancing effect of *Atractylodes lancea* rhizome. J Ethnopharmacol. (1990) 29:341–4. 10.1016/0378-8741(90)90044-T 2214818

[2] Koonrungsesomboon N Na-Bangchang K Karbwang J. Therapeutic potential and pharmacological activities of *Atractylodes lancea* (Thunb.) DC. Asian Pac J Trop Med. (2014) 7:421–8. 10.1016/S1995-7645(14)60069-9 25066389

[3] Liu C Song C Wang Y Xiao Y Zhou Z Cao G. Deep-fried *Atractylodes lancea* rhizome alleviates spleen deficiency diarrhea-induced short-chain fatty acid metabolic disorder in mice by remodeling the intestinal flora. J Ethnopharmacol. (2023) 303:115967. 10.1016/j.jep.2022.115967 36442762

[4] Xie Y Zhan X Tu J Xu K Sun X Liu C. Atractylodes oil alleviates diarrhea-predominant irritable bowel syndrome by regulating intestinal inflammation and intestinal barrier via SCF/c-kit and MLCK/MLC2 pathways. J Ethnopharmacol. (2021) 272:113925. 10.1016/j.jep.2021.113925 33592255

[5] Chen MH May BH Zhou IW Zhang AL Xue CC. Integrative medicine for relief of nausea and vomiting in the treatment of colorectal cancer using oxaliplatin-based chemotherapy: a systematic review and meta-analysis. Phytother Res. (2016) 30:741–53. 10.1002/ptr.5586 26912094

[6] Zhu XY Cheng GL Liu FH Yu J Wang YJ Yu TQ. Taguchi approach for anti-heat stress prescription compatibility in mice spleen lymphocytes *in vitro*. Arch Pharm Res. (2011) 34:1125–33. 10.1007/s12272-011-0710-2 21811919

[7] Liu C Wang S Xiang Z Xu T He M Xue Q. The chemistry and efficacy benefits of polysaccharides from *Atractylodes macrocephala* Koidz. Front Pharmacol. (2022) 13:952061. 10.3389/fphar.2022.952061 36091757 PMC9452894

[8] Chu H Zong Y Yang H Chen S Ma Z Li H. Effects of Yu-Ping-Feng polysaccharides on animal growth performance and immune function: a review. Front Vet Sci. (2023) 10:1260208. 10.3389/fvets.2023.1260208 37799408 PMC10547873

[9] Zhu B Zhang QL Hua JW Cheng WL Qin PL. The traditional uses, phytochemistry, and pharmacology of Atractylodes macrocephala Koidz: a review. J Ethnopharmacol. (2018) 226:143–67. 10.1016/j.jep.2018.08.023 30130541

[10] Ruqiao L Yueli C Xuelan Z Huifen L Xin Z Danjie Z. Rhizoma *Atractylodis macrocephalae*: a review of photochemistry, pharmacokinetics and pharmacology. Pharmazie. (2020) 75:42–55. 10.1691/ph.2020.9738 32213234

[11] Wang Y Li X Jiang Q Sun H Jiang J Chen S. GC-MS analysis of the volatile constituents in the leaves of 14 compositae plants. Molecules. (2018) 23:166. 10.3390/molecules23010166 29346294 PMC6016956

[12] Wang X Li L Ran X Dou D Li B Yang B. What caused the changes in the usage of *Atractylodis macrocephalae* Rhizoma from ancient to current times? J Nat Med. (2016) 70:36–44. 10.1007/s11418-015-0934-4 26382100

[13] Seo HY Kim MK Lee SH Hwang JSK Park G Jang KB. Kahweol ameliorates the liver inflammation through the inhibition of NF-κB and STAT3 activation in primary Kupffer cells and primary hepatocytes. Nutrients. (2018) 10:863. 10.3390/nu10070863 29973533 PMC6073512

[14] Yu Z Xu S-F Zhao J-L Zhao L Zhang A-Z Li M-Y. Toxic effects of hexavalent chromium (Cr6+) on bioaccumulation, apoptosis, oxidative damage and inflammatory response in *Channa asiatica*. Environ Toxicol Pharmacol. (2021) 87:103725. 10.1016/j.etap.2021.103725 34416396

[15] Zhao L Zhao JL Bai Z Du J Shi Y Wang Y. Polysaccharide from dandelion enriched nutritional composition, antioxidant capacity, and inhibited bioaccumulation and inflammation in *Channa asiatica* under hexavalent chromium exposure. Int J Biol Macromol. (2022) 201:557–68. 10.1016/j.ijbiomac.2021.12.117 35007636

[16] Li CQ He LC Jin QJ. Atractylenolide I and atractylenolide III inhibit Lipopolysaccharide-induced TNF-α and NO production in macrophages. Phytother Res. (2007) 21:347–53. 10.1002/ptr.2040 17221938

[17] Jeong D Dong G-Z Lee HJ Ryu J-H. Anti-inflammatory compounds from *Atractylodes macrocephala*. Molecules. (2019) 24:1859. 10.3390/molecules24101859 31091823 PMC6571718

[18] Kim J-H. Polyacetylenic compounds from *Atractylodes rhizomes*. Korea J Herbol. (2016) 31:25–39. 10.6116/kjh.2016.31.5.25

[19] Apel K Hirt H. Reactive oxygen species: metabolism, oxidative stress, and signal transduction. Annu Rev Plant Biol. (2004) 55:373–99. 10.1146/annurev.arplant.55.031903.141701 15377225

[20] Li X Lin J Han W Mai W Wang L Li Q. Antioxidant ability and mechanism of rhizoma *Atractylodes macrocephala*. Molecules. (2012) 17:13457–72. 10.3390/molecules171113457 23149564 PMC6268131

[21] Yu M Chen L Peng Z Wang D Song Y Wang H. Embryotoxicity caused by DON-induced oxidative stress mediated by Nrf2/HO-1 pathway. Toxins. (2017) 9:188. 10.3390/toxins9060188 28598396 PMC5488038

[22] Han B Gao Y Wang Y Wang L Shang Z Wang S. Protective effect of a polysaccharide from *Rhizoma atractylodis* macrocephalae on acute liver injury in mice. Int J Biol Macromol. (2016) 87:85–91. 10.1016/j.ijbiomac.2016.01.086 26820352

[23] Lee J-H Van ND Ma J-Y Kim Y-B Kim S-K Paik H-D. Screening of antiviral medicinal plants against avian influenza virus H1N1 for food safety. Food Sci Anim Resour. (2010) 30:345–50. 10.5851/kosfa.2010.30.2.345

[24] Shah A Krishnamurthy R. Swine flu and its herbal remedies. Int J Eng Sci. (2013) 2:68–78. 10.5958/0974-360X.2016.00362.0

[25] Shi S Qin Z Kong S Lai X Su Z Zeng H. Effective component selection of atraotydin extract against influenza virus. Shih-chen Kuo I Kuo Yao. (2012) 23:565–6.

[26] Cheng Y Mai JY Hou TL Ping J Chen JJ. Antiviral activities of atractylon from *Atractylodis rhizoma*. Mol Med Rep. (2016) 14:3704–10. 10.3892/mmr.2016.5713 27600871 PMC5042776

[27] Yang L Yu H Hou A Man W Wang S Zhang J. A review of the ethnopharmacology, phytochemistry, pharmacology, application, quality control, processing, toxicology, and pharmacokinetics of the dried rhizome of *Atractylodes macrocephala*. Front Pharmacol. (2021) 12:727154. 10.3389/fphar.2021.727154 34803677 PMC8595830

[28] Luo L Cai J Zhou Z Tang W Xue J Liu J. Polysaccharides from *Rhizoma atractylodis* macrocephalae: a review on their extraction, purification, structure, and bioactivities. Evid Bas Complement Alternat Med. (2022) 2022:2338533. 10.1155/2022/2338533 36034948 PMC9402290

[29] Yu Z Zhao L Zhao J-L Xu W Guo Z Zhang A-Z. Dietary *Taraxacum mongolicum* polysaccharide ameliorates the growth, immune response, and antioxidant status in association with NF-κB, Nrf2 and TOR in Jian carp (*Cyprinus carpio* var. Jian) *Aquaculture*. (2022) 547:737522. 10.1016/j.aquaculture.2021.737522

[30] Wang J-H Bose S Kim H-G Han K-S Kim H. Fermented *Rhizoma atractylodis* macrocephalae alleviates high fat diet-induced obesity in association with regulation of intestinal permeability and microbiota in rats. Sci Rep. (2015) 5:8391. 10.1038/srep08391 25684573 PMC4329570

[31] Li L Wu X Peng H Fan M Hou Z Kong X. The effect of dietary addition of a polysaccharide from Atractylodes macrophala Koidz on growth performance, immunoglobulin concentration and IL-1β expression in weaned piglets. J Agric Sci. (2009) 147:625–31. 10.1017/S002185960999013X

[32] Xu D Li B Cao N Li W Tian Y Huang Y. The protective effects of polysaccharide of *Atractylodes macrocephala* Koidz (PAMK) on the chicken spleen under heat stress via antagonizing apoptosis and restoring the immune function. Oncotarget. (2017) 8:70394. 10.18632/oncotarget.19709 29050288 PMC5642563

[33] Xu C Zhao Y Shang X Niu W. The effects of supplementing diets with *Atractylodes macrocephala* Koidz rhizomes on growth performance and immune function in piglets. J Anim Feed Sci. (2012) 21:302–11. 10.22358/jafs/66078/2012

[34] Hu W Huang K Zhang L Ni J Xu W Bi S. Immunomodulatory effect of *Atractylodis macrocephala* Koidz. polysaccharides *in vitro*. Poult Sci. (2024) 103:103171. 10.1016/j.psj.2023.103171 37925772 PMC10652128

[35] Xu ZhenSong XZ Qu MingRen MQ Song XiaoZhen XS Zhao XiangHui XZ Huang Tao TH Chen YuMin YC. Effects of *Rhizoma atractylodis* oil on rumen fermentation and nutrient degradability *in vitro* in Jinjiang yellow cattle. Chin J Anim Nutri. (2014) 26:2373–8. 10.5555/20143326627

[36] Li L Yin F Zhang B Peng H Li F Zhu N. Dietary supplementation with *Atractylodes macrophala Koidz polysaccharides* ameliorate metabolic status and improve immune function in early-weaned pigs. Livest Sci. (2011) 142:33–41. 10.1016/j.livsci.2011.06.013

[37] Wang X Wang Y Mao Y Hu A Xu T Yang Y. The beneficial effects of traditional Chinese medicine on antioxidative status and inflammatory cytokines expression in the liver of piglets. Front Vet Sci. (2022) 9:937745. 10.3389/fvets.2022.937745 36213414 PMC9539681

[38] Li W Guo S Xu D Li B Cao N Tian Y. Polysaccharide of *Atractylodes macrocephala* Koidz (PAMK) relieves immunosuppression in cyclophosphamide-treated geese by maintaining a humoral and cellular immune balance. Molecules. (2018) 23:932. 10.3390/molecules23040932 29673208 PMC6017956

[39] Xu W Guan R Shi F Du A Hu S. Structural analysis and immunomodulatory effect of polysaccharide from *Atractylodis macrocephalae* Koidz on *Bovine lymphocytes*. Carbohydr Polym. (2017) 174:1213–23. 10.1016/j.carbpol.2017.07.041 28821047

[40] Bose S Kim H. Evaluation of *in vitro* anti-inflammatory activities and protective effect of fermented preparations of Rhizoma Atractylodis Macrocephalae on intestinal barrier function against lipopolysaccharide insult. Evid Bas Complement Alternat Med. (2013) 2013:363076. 10.1155/2013/363076 23573125 PMC3612467

[41] Shi K Qu L Lin X Xie Y Tu J Liu X. Deep-fried atractylodis rhizoma protects against spleen deficiency-induced diarrhea through regulating intestinal inflammatory response and gut microbiota. Int J Mol Sci. (2019) 21:124. 10.3390/ijms21010124 31878055 PMC6981650

[42] Li W Xiang X Li B Wang Y Qian L Tian Y. PAMK Relieves LPS-induced enteritis and improves intestinal flora disorder in goslings. Evid Bas Complement Alternat Med. (2021) 2021:9721353. 10.1155/2021/9721353 33688370 PMC7920704

[43] Wang R Zhou G Wang M Peng Y Li X. The metabolism of polysaccharide from *Atractylodes macrocephala* Koidz and its effect on intestinal microflora. Evid Bas Complement Alternat Med. (2014) 2014:926381. 10.1155/2014/926381 25505927 PMC4258363

[44] Wang SP Wang WJ Tan ZL Liu GW Zhou CF Yin J. Effect of traditional Chinese medicine compounds on rumen fermentation, methanogenesis and microbial flora *in vitro*. Anim Nutr. (2019) 5:185–90. 10.1016/j.aninu.2018.09.004 31193871 PMC6544579

[45] Xu W Fang S Wang Y Chi X Ma X Zhang T. Receptor and signaling pathway involved in bovine lymphocyte activation by *Atractylodis macrocephalae* polysaccharides. Carbohydr Polym. (2020) 234:115906. 10.1016/j.carbpol.2020.115906 32070525

[46] Pedersen KS Kristensen CS Nielsen PJ. Demonstration of non-specific colitis and increased crypt depth in colon of weaned pigs with diarrhea. Vet Quart. (2012) 32:45–9. 10.1080/01652176.2012.675091 22469034

[47] Kai L Zong X Jiang Q Lu Z Wang F Wang Y. Protective effects of polysaccharides from *Atractylodes macrocephalae* Koidz against dextran sulfate sodium induced intestinal mucosal injury on mice. Int J Biol Macromol. (2022) 195:142–51. 10.1016/j.ijbiomac.2021.12.042 34896465

[48] Wei W Li H Deng Y Zheng X Zhou Y Xue X. The combination of Alisma and *Atractylodes ameliorates* cerebral ischaemia/reperfusion injury by negatively regulating astrocyte-derived exosomal miR-200a-3p/141-3p by targeting SIRT1. J Ethnopharmacol. (2023) 313:116597. 10.1016/j.jep.2023.116597 37146842

[49] Yang M Zhang Q Taha R Abdelmotalab MI Wen Q Yuan Y. Polysaccharide from *Atractylodes macrocephala* Koidz. ameliorates DSS-induced colitis in mice by regulating the Th17/Treg cell balance. Front Immunol. (2022) 13:1021695. 10.3389/fimmu.2022.1021695 36341374 PMC9630481

[50] Zhang Q Deng Y Wang J Haung F Zhou Y Jia M. Efficacy and safety of Shenling Atractylodes Powder in the treatment of ulcerative colitis: a protocol for systematic review and meta-analysis. Medicine. (2021) 100:e25355. 10.1097/MD.0000000000025355 33832115 PMC8036058

[51] Qu L Shi K Xu J Liu C Ke C Zhan X. Atractylenolide-1 targets SPHK1 and B4GALT2 to regulate intestinal metabolism and flora composition to improve inflammation in mice with colitis. Phytomedicine. (2022) 98:153945. 10.1016/j.phymed.2022.153945 35114452

[52] Bagath M Krishnan G Devaraj C Rashamol VP Pragna P Lees AM. The impact of heat stress on the immune system in dairy cattle: a review. Res Vet Sci. (2019) 126:94–102. 10.1016/j.rvsc.2019.08.011 31445399

[53] Vandana GD Sejian V Lees AM Pragna P Silpa MV Maloney K. Heat stress and poultry production: impact and amelioration. Int J Biometeorol. (2021) 65:163–79. 10.1007/s00484-020-02023-7 33025116

[54] Chen S Yong Y Ju X. Effect of heat stress on growth and production performance of livestock and poultry: mechanism to prevention. J Therm Biol. (2021) 99:103019. 10.1016/j.jtherbio.2021.103019 34420644

[55] Guo KJ Xu SF Yin P Wang W Song XZ Liu FH. Active components of common traditional Chinese medicine decoctions have antioxidant functions. J Anim Sci. (2011) 89:3107–15. 10.2527/jas.2010-3831 21571894

[56] José Karpeggiane de Oliveira M Diego Brandão Melo A Alves Marçal D Alves da Cunha Valini G Alisson Silva C Mari Veira A. Effects of lowering dietary protein content without or with increased protein-bound and feed-grade amino acids supply on growth performance, body composition, metabolism, and acute-phase protein of finishing pigs under daily cyclic heat stress. J Anim Sci. (2023) 101:skac387. 10.1093/jas/skac387 36420675 PMC9833036

[57] Xu D Li W Li B Tian Y Huang Y. The effect of selenium and polysaccharide of *Atractylodes macrocephala* koidz. (PAMK) on endoplasmic reticulum stress and apoptosis in chicken spleen induced by heat stress. RSC Adv. (2017) 7:7519–25. 10.1039/C6RA27730F

[58] Chen Y Wu Y Wang H Gao K. A new 9-nor-atractylodin from *Atractylodes lancea* and the antibacterial activity of the atractylodin derivatives. Fitoterapia. (2012) 83:199–203. 10.1016/j.fitote.2011.10.015 22061661

[59] Peng W Han TW Xin B Zhang XG Zhang QY Jia M. Comparative research of chemical constituents and bioactivities between petroleum ether extracts of the aerial part and the rhizome of A*tractylodes macrocephala*. Med Chem Res. (2011) 20:146–51. 10.1007/s00044-010-9311-8

[60] Ohara K Katayama M Nagai K. β-Eudesmol, an oxygenized sesquiterpene, affects efferent adrenal sympathetic nerve activity via transient receptor potential ankyrin 1 in rats. Neurosci Lett. (2018) 684:18–24. 10.1016/j.neulet.2018.06.057 29966754

[61] More S Choi D-K. Neuroprotective role of atractylenolide-I in an *in vitro* and *in vivo* model of Parkinson's disease. Nutrients. (2017) 9:451. 10.3390/nu9050451 28468332 PMC5452181

[62] Zhang WJ Zhao ZY Chang LK Cao Y Wang S Kang CZ., et al. Atractylodis rhizoma: a review of its traditional uses, phytochemistry, pharmacology, toxicology and quality control. J Ethnopharmacol. (2021 266:113415. 10.1016/j.jep.2020.113415 32987126 PMC7521906

[63] Jeong YH Li W Go Y Oh Y-C. Atractylodis rhizoma alba attenuates neuroinflammation in BV2 microglia upon LPS stimulation by inducing HO-1 activity and inhibiting NF-κB and MAPK. Int J Mol Sci. (2019) 20:4015. 10.3390/ijms20164015 31426492 PMC6720582

[64] Oladokun S Adewole ID. Biomarkers of heat stress and mechanism of heat stress response in Avian species: current insights and future perspectives from poultry science. J Therm Biol. (2022) 110:103332. 10.1016/j.jtherbio.2022.103332 36462852

